# Smoking and finances: baseline characteristics of low income daily smokers in the FISCALS cohort

**DOI:** 10.1186/s12939-017-0643-6

**Published:** 2017-08-30

**Authors:** Kristy A. Martire, Philip Clare, Ryan J. Courtney, Billie Bonevski, Veronica Boland, Ron Borland, Christopher M. Doran, Michael Farrell, Wayne Hall, Jaimi M. Iredale, Mohammad Siahpush, Richard P. Mattick

**Affiliations:** 1 School of Psychology, University of NSW, Sydney, NSW 2052 Australia; 2 National Drug and Alcohol Research Centre, University of NSW, Sydney, NSW 2052 Australia; 3 School of Medicine & Public Health, University of Newcastle, University Dr, Callaghan, NSW 2308 Australia; 4 Centre for Behavioural Research in Cancer, Cancer Council Victoria, 615 St Kilda Rd, Melbourne, VIC 3004 Australia; 5 Centre for Indigenous Health Equity Research, School of Human, Health and Social Sciences, Central Queensland University, Brisbane, Australia; 6 Centre for Clinical Research, University of Queensland, Brisbane, Australia; 7 College of Public Health, University of Nebraska Medical Centre, 42nd and Emile, Omaha, NE 68198 USA

**Keywords:** Financial stress, Socioeconomic status, Tobacco, Smoking, Low-income

## Abstract

**Background:**

Financial stress is a barrier to successful smoking cessation and a key predictor of relapse. Little is known about the financial situation of low-income Australian daily smokers. This study aims to describe and investigate associations between the financial functioning, tobacco use and quitting behaviours of low income daily smokers.

**Methods:**

Low-income Australian adult smokers in the ‘Financial Intervention for Smoking Cessation Among Low-income Smokers (FISCALS) randomised clinical trial completed a structured telephone questionnaire.

**Results:**

The median number of cigarettes typically smoked by the 1047 participants was 23 per day. The median spent on tobacco per week was AU$80. Three quarters (73.0%) reported some financial stress and 43.2% reported smoking-induced deprivation. Financial stress was significantly associated with deprivation (IRR: 1.23, 95% CI 1.21, 1.26, *p* < 0.001). There were no significant associations either between adjusted financial stress or deprivation and motivation to quit or certainty of quit success.

**Conclusions:**

Financial stress and smoking induced deprivation were prevalent among low-income daily smokers, but they were not associated with motivation to quit. Smoking cessation interventions need to be responsive to the role financial stress plays in reducing quit attempts and increasing relapse.

**Trial registration:**

Australian and New Zealand Clinical trials Registry ACTRN12612000725864 6/07/2012

**Electronic supplementary material:**

The online version of this article (10.1186/s12939-017-0643-6) contains supplementary material, which is available to authorized users.

## Background

Australians from disadvantaged areas are three times more likely to smoke daily than those from the most advantaged areas (20% *c.f.* 7%) [1]. This uneven distribution of tobacco use [2, 3] and cessation [4] by socioeconomic status (SES; or its proxies) is also observed in other high income countries. As a consequence of this, proportionally larger reductions in smoking rates are needed among disadvantaged groups if national targets for smoking prevalence are to be achieved [5–7].

Tobacco taxation is an effective strategy to reduce smoking rates in low SES population groups [3, 8, 9]. However, financial stress has been identified as a barrier to successful cessation and a key predictor of smoking relapse [10, 11]. There is a pernicious cycle where tobacco use increases financial pressures (e.g., difficulty paying for bills, food, rent etc.) [12], which in turn maintains smoking [13], by the use of price-minimization strategies (e.g., by switching to roll-your-own), rather than cessation [14]. This may mean that price-based interventions will deliver diminishing returns over time with socially disadvantaged smokers because rises in taxation increase financial stress and smoking, and decrease quit success [15].

Cross-sectional and longitudinal evaluation studies have indicated that Australian smokers vary in their SES and motivations, intentions, and timeframes for quitting (e.g., The International Tobacco Control (ITC) Four-Country Study, The National Drug Strategy Household Survey; and the Household, Income & Labour Dynamics in Australia (HILDA) survey). However, these data sources do not bring together detailed information about financial functioning, tobacco use and quitting. This was one of the aims of the Financial Intervention for Smoking Cessation Among Low-income Smokers (FISCALS) Randomised Clinical Trial (RCT). The 1047 FISCALS smokers represent a large sample of Australian daily smokers selected on the basis of their low-income (defined as being in receipt of a means-tested government pension or allowance) and their willingness to quit within one-month of trial enrolment. Their data provides novel insights into the interplay between financial stress, smoking and cessation that are necessary to guide the development and delivery of effective tobacco control interventions for disadvantaged smokers. This is essential if cigarette tax increases provide either diminishing returns in the future [15], or adversely affect low SES smokers who are unable to quit.

The aim of this paper was to describe and investigate associations between the financial functioning, tobacco use and quitting behaviours of low SES smokers on entry to the FISCALS trial.

## Methods

The primary aim of the FISCALS RCT was to evaluate the efficacy of a financial education and support program (FESP) in improving smoking cessation rates among low SES smokers. The detailed protocol of the FISCALS RCT has been reported elsewhere [16]. This study reports only data collected at baseline. Key eligibility criteria for recruitment into FISCALS were: 1) being aged 18 years or older; 2) being in receipt of a government pension or allowance; 3) smoking more than 10 cigarettes per day; 4) being interested in quitting, and; 5) being willing to make a quit attempt within 1 month of recruitment. Participants were recruited to the trial between April, 2013 and September, 2014 via Quitlines, community newspaper advertisements, and Australian Government Department of Human Services Centrelink Customer Service Centres.

### Survey instruments

Survey items were used to collect demographic information and to examine type and price of tobacco purchased, motivation to quit, methods used for past cessation attempts, financial functioning, financial stress, deprivation and smoking induced deprivation. Where possible we used validated measures and those previously appearing in the literature. (See Tables 2 and 3 for further information regarding response options).

#### Tobacco use and quitting behaviours

##### Tobacco use

We assessed typical number of cigarettes smoked per day, type of tobacco, weekly tobacco expenditure, and length of last quit attempt (in days).

##### Nicotine dependence

We calculated a Heaviness of Smoking Index (HSI) [17] score for each participant, based on the number of cigarettes smoked and the time until first cigarette each day. Scores range from 0 to 6, with scores of 4 or more indicating ‘heavy smokers’ [18]. We also assessed frequency of strong urges to smoke with the question: “How often do you get strong urges to smoke?”

##### Quitting behaviours

We assessed past quit attempts with the question: “Have you ever tried to quit smoking?” We determined participants’ confidence in the success of their next quit attempt by asking: “If you decided to give up smoking completely, how sure are you that you would succeed?” We assessed motivation to quit smoking with the question “How would you rate your current motivation to give up smoking”.

#### Methods used for smoking cessation

##### NRT and other pharmacotherapy use

We asked participants: “Prior to entering the study, had you ever used Nicotine Replacement Therapy (NRT)?” and “Have you ever used prescription medications to help you quit smoking”. If yes, we then asked participants to specify the types of medications used. We also assessed perceptions of past and future NRT use with the questions: “On your last quit attempt using NRT, how effective did you find NRT to help you quit smoking?” and “How much do you think NRT will increase your chances of successfully quitting in your current quit attempt?”

##### Quitline use

We determined past use of Quitline services by asking: “Prior to entering this study, had you ever spoken to the Quitline?”

#### Financial functioning

We collected information on weekly household income. Participants were asked: “Have you ever sought professional help with managing your finances?” If yes, they were asked to specify if this was within the past 12 months.

##### Financial stress

We assessed financial stress using seven of the eight items from the Financial Stress Inventory (FSI) [19]. The FSI requires participants to indicate: “In the last month did any of the following happen to you because of a lack of money” and then to select all of the following that apply: a) could not pay electricity, gas or telephone bills on time; b) could not pay the mortgage or rent on time; c) pawned or sold something; d) went without meals; e) was unable to heat the home; f) asked for financial help from friends or family; g) asked for help from a welfare or community organisation. Scores ranged from 0 to 7 with anything greater than 0 indicating the presence of financial stress.

##### Deprivation

We measured deprivation using six questions developed by the Australian Bureau of Statistics for the Household Expenditure Survey [20]. Participants were asked to identify if any of the following list of activities applied to them: a) Have a holiday away from home for at least 1 week a year; b) Have a night out once a fortnight; c) Have friends or family over for a meal once a month; d) Have a special meal once a week; e) Buy new and not second hand clothes, most of the time; f) Spend time on leisure or hobby activities. If no, participants were then asked to indicate for each item: “Is the reason that you [don’t have a holiday away from home for at least one week a year] because you: a) don’t want to, b) can’t afford to, or c) some other reason?” with the text in brackets reflecting the relevant item. The scale was constructed by adding the number of endorsed items, leading to a score in the range of 0–6.

##### Smoking induced deprivation (SID)

We measured SID in two ways. Presence or absence was assessed with the question: “In the past month, has there been a time when the money you spent on cigarettes resulted in not having enough money for household essential such as food?” (yes/no). The International Tobacco Control survey uses the same question using a six-month window [21]. The second (categorical) measure of SID asked participants to indicate their agreement with the statement: “Spending money on cigarettes means missing out on things that are important to me” using a five-point Likert scale from ‘strongly disagree’ to ‘strongly agree’.

#### Relative socio-economic advantage and disadvantage

We measured the relative socio-economic advantage or disadvantage of participants using the Socio-Economic Indexes for Areas (SEIFA) [22]. Areas are divided into five quintiles; the top quintile represents the most advantaged and the bottom quintile represents the most disadvantaged.

### Data analysis

We conducted all analyses using Stata 14.1 [23]. Demographic information, tobacco use, quitting behaviours and financial functioning were analysed descriptively. Categorical variables were presented as number (%). None of the ordinal or continuous variables were normally distributed based on Shapiro-Wilk normality tests, so are presented as median and interquartile range (IQR).

Financial stress (FSI) and financial deprivation scores were analyzed for associations with motivation to quit and certainty of quit success. Because FSI and financial deprivation scores are count variables with no over-dispersion, analysis was conducted using Poisson regression, with results presented as incidence-rate ratios (IRR). Analyses were conducted both unadjusted, and adjusted for potential confounding by age, sex, education, SEIFA and HSI. Type of tobacco used (factory made cigarettes or roll-your-own) was examined for associations with a range of financial functioning indicators: a) ability to pay bills on time (single-item FSI measure) using χ^2^; b) weekly tobacco expenditure (Mann-Whitney U); c) proportion of household income spent on tobacco products (Kruskal-Wallis H); d) FSI score (Mann-Whitney U), and e) deprivation (χ^2^). To control the Type I error rate due to the number of analyses conducted, we used a Bonferonni-adjusted critical *p*-value of *p* = 0.004 to assess statistical significance.

## Results

### Demographic information

The RCT participation rate was 40.9% giving a final sample size of 1047 participants (*SI Trial Enrolment* (Additional file 1)). Almost half of the sample was recruited from New South Wales (49.7%), one quarter from Queensland (24.4%), and approximately one fifth from Victoria (14%). See Table 1 for further information about the demographic characteristics of the study participants.

**Table 1:** Demographic characteristics of the study participants (*N* = 1047)

	Number (%) or median (IQR)
Age, median (IQR) years	46 (35–56)
Sex, female	557 (53.2%)
Aboriginal or Torres Strait Islander	70 (6.7%)
Marital status	
Married/partnered/de-facto	319 (30.5%)
Single/never married	359 (34.3%)
Separated/divorced/widowed	366 (35.0%)
Current living arrangements	
Single-person household	442 (42.2%)
Two or more persons	604 (57.7%)
Including one or more child	375 (35.8%)
Government pensions or allowance^a^	
Age pension	95 (9.2%)
Newstart Allowance	288 (27.5%)
Disability Support Pension	352 (33.6%)
Parenting Payment	128 (12.2%)
Family tax benefit A or B	198 (18.9%)
Other	201 (19.2%)
Education	
Commenced/completed primary school	9 (0.9%)
Commenced/completed secondary school	650 (62.9%)
Technical or further education	281 (26.8%)
Some University at least	100 (9.6%)
Employment^b^	
Employed fulltime/part-time/casual	160 (15.3%)
Unemployed/home duties/retired	628 (60.0%)
Unable to work	328 (31.3%)
Student	74 (7.1%)
Diagnosed or treated in past 12 months	
Depression	478 (45.7%)
Anxiety	364 (34.8%)
Schizophrenia/psychosis	103 (9.8%)
Bipolar disorder	91 (8.7%)
Personality disorder	53 (5.1%)
PTSD	33 (3.2%)
Other	93 (8.9%)
DASS-21^c^	
Depression, median (IQR)	14 (6–26)
Anxiety, median (IQR)	12 (4–22)
Stress, median (IQR)	18 (10–28)

^a^Some participants were in receipt of more than one benefit type. In order, briefly characterized these benefits are financial support for: some older Australians; those looking for work or participating in approved activities that may increase employability; people who have a physical, intellectual or psychiatric condition that stops them from working; parents or guardians raising children

^b^Some participants selected more than one employment descriptor

^c^Depression Anxiety Stress Scales [31]

### Tobacco use

The median number of cigarettes participants typically smoked was 23 per day (IQR 15–30) (see Table 2), and they first started smoking at 15 years (IQR 14–18). Most reported smoking mainly or only factory-made cigarettes (60.6%) purchased from the supermarket (72.0%).

**Table 2:** Tobacco use and Quit Methods

	Number (%) or median (IQR)
Tobacco use (*N* = 1047)	
Cigarettes per day	23 (15–30)
Heaviness of Smoking Index	4 (3–5)
Type of tobacco smoked	
Mainly/only factory made	634 (60.6%)
Factory made and RYO^a^ equally	96 (9.2%)
Mainly/only RYO	314 (30.0%)
Frequency of strong urges	
Never	18 (1.7%)
Daily or less	74 (7.1%)
Several times daily	320 (30.6%)
Hourly or more	630 (60.2%)
Ever attempted to quit, yes	970 (92.6%)
Days since last quit attempt^b^	365 (122–1096)
Length of last quit attempt, days^c^	7 (3–21)
Confidence in success of next quit attempt	
Not sure of success	75 (7.2%)
Slightly sure	100 (9.6%)
Moderately sure	342 (32.7%)
Very sure	325 (31.0%)
Extremely sure	176 (16.8%)
Current motivation to quit	8 (7–10)
Methods used for past quit attempts (*N* = 970**)**	
Ever used Nicotine Replacement Therapy, yes	684 (70.5%)
NRT Type used on last quit attempt^d,e^	
Gum	161 (23.5%)
Patch	523 (76.5%)
Lozenge	81 (11.8%)
Inhaler	73 (10.7%)
Sublingual tablets/ Mouth spray/mist/e-cigarette	53 (7.7%)
Ever used prescription medications, yes	478 (49.3%)
Prescription medications used^e,f^	
Burpropion	143 (29.9%)
Varenicline	415 (86.8%)
Other	5 (1.1%)
Ever called a Quitline, yes	368 (37.6%)

^a^RYO = Roll-your-own

^b^ *n* = 966

^c^Only includes those who attempted to quit in past year (*n* = 378)

^d^ *n* = 684

^e^Some participants used more than one type of quit support

^f^ *n* = 478

Three fifths of participants (60.2%) said that they experienced strong cravings hourly or more and nearly all had made a previous quit attempt (92.6%), with 378 (36.1%) doing so in the past year, for a median of 7 days (IQR 3–21).

### Methods used to quit smoking

Almost three quarters of participants had used NRT in a past quit attempt (70.5%), almost half had used prescription medications (49.3%). More than a third had called a Quitline (37.6%) (See Table 2).

Among those who had used NRT, 76.5% used patches. Of the 684 participants who reported NRT use, 67.3% considered it had been at least ‘moderately’ effective. Almost three quarters of the total sample (73.4%) thought NRT would increase their chances of quit success in their next attempt ‘very much’ or ‘extremely’. For most, confidence in future quit success was at least moderate (80.5%), and motivation to quit smoking was high (median 8/10, IQR 7–10).

### Financial functioning

Overall, 27% of the sample lived within the most disadvantaged SEIFA quintile [22], 27.7% were from the second most disadvantaged quintile. The majority (62.9%) reported a weekly household income of less than $579 per week ($30,108 p.a.) (See Table 3).

**Table 3:** Financial functioning (*N* = 1047)

	Number (%) or median (IQR)
Weekly household income	
< $190	30 (2.9%)
$190–$379	341 (32.6%)
$380–$579	287 (27.4%)
$580–$769	146 (13.9%)
> $770	243 (23.2%)
Ever sought professional help managing finances, yes	257 (24.6%)
Past 12 months	112 (10.7%)
Financial stress in past month (0≤FSI^a^≤7)	2 (0–4)
Any financial stress in past month (FSI^a^ >0)	764 (73.0%)
FSI items endorsed for past month	
Difficulty paying electricity/gas/telephone bills	424 (40.5%)
Difficulty paying mortgage or rent on time	168 (16.1%)
Pawned or sold something	291 (27.8%)
Went without meals	307 (29.3%)
Unable to heat the home	146 (13.9%)
Asked for financial help from friends or family	550 (52.5%)
Asked for help from welfare or community group	350 (33.4%)
Financial deprivation^b^, no	
Have holiday away for at least 1 week per year	566 (54.1%)
Have night out once a fortnight	407 (38.9%)
Host a meal once a month	145 (13.8%)
Have special meal once a week	242 (23.1%)
Buy new clothes most of the time	320 (30.6%)
Spend time on leisure or hobbies	290 (27.7%)
Weekly tobacco expenditure	$80 ($50–$120)
Smoking induced deprivation past month, yes	452 (43.2%)
Make ‘important’ sacrifices to buy tobacco	
Strongly disagree	22 (2.1%)
Disagree	171 (16.5%)
Neutral	51 (4.9%)
Agree	546 (52.1%)
Strongly agree	245 (23.6%)

^a^FSI Financial Stress Index

^b^Data only for those who reported that they could not afford the activity

Almost three quarters of participants (73.0%) reported some financial stress as measured by the FSI and 10.6% of all participants had sought professional help to manage their finances in the past 12 months. Those who were more motivated to quit reported higher unadjusted financial stress (IRR: 1.04; 95% CI 1.01, 1.06, *p* = 0.003) but not when adjusting for other covariates (IRR: 1.03; 95% CI 1.01, 1.06, *p* = 0.005), as seen in Table 4. There was no significant association between certainty of quit success and financial stress score, either unadjusted (IRR: 1.02, 95% CI 0.99, 1.06, *p* = 0.220) or adjusted (IRR: 1.04, 95% CI 1.00, 1.08, *p* = 0.061).

**Table 4:** Unadjusted and adjusted associations between motivation to quit and certainty of quit success, and Financial Stress Inventory and Deprivation

		Financial Stress Scale		Deprivation	
		IRR (95% CI)	*p*-value	IRR (95% CI)	*p*-value
Motivation to Quit	Unadjusted^a^	1.04 (1.01, 1.06)	*p* = 0.003	1.04 (1.01, 1.06)	*p* = 0.004
	Adjusted^b^	1.03 (1.01, 1.06)	*p* = 0.005	1.03 (1.00, 1.05)	*p* = 0.030
Certainty of Quit Success	Unadjusted^a^	1.02 (0.99, 1.06)	*p* = 0.220	1.03 (0.99, 1.08)	*p* = 0.101
	Adjusted^b^	1.04 (1.00, 1.08)	*p* = 0.061	1.04 (1.00, 1.09)	*p* = 0.041

^a^Bivariate model with single predictor/outcome. ^b^ Multivariate model controlling for age, sex, education, SEIFA and HSI

Deprivation was prevalent with between 13.8 and 54.1% of participants reporting that they could not afford to engage in the activities listed. Unlike FSI, there were no significant differences in deprivation based on motivation to quit, either unadjusted (IRR: 1.04; 95% CI 1.01, 1.06, *p* = 0.004), or adjusted (IRR: 1.03; 95% CI 1.00, 1.05, *p* = 0.030). Similarly, there was no significant association between certainty of quit success and deprivation, either unadjusted (IRR: 1.03, 95% CI 0.99, 1.08, *p* = 0.101) or adjusted (IRR: 1.04, 95% CI 1.00, 1.09, *p* = 0.041). Financial stress was significantly associated with deprivation (IRR: 1.23, 95% CI 1.21, 1.26, *p* < 0.001).

The first question of the FSI has been used as a measure of financial stress in past research [17]. This item was endorsed by 40.5% of the sample and was significantly associated with the type of cigarette smoked: those who smoked FMC only were more likely to report not paying bills on time (42.1%) than those who smoked RYO only (35.3%; χ^2^(2) = 12.889, *p* = 0.002).

The median reported weekly tobacco expenditure in the sample was $80 per week (range $10–$300, IQR $50–$100). Those who smoked only FMC spent significantly more on cigarettes per week (Median $100; IQR $75–$130) than those who smoked only RYO (Median $50; IQR $40–$75; *n* = 783, z = 13.61, *p* < 0.001).

Of relevance to the calculation of household tobacco expenditure, almost half of participants (42.2%) resided in single smoker households, living either alone (29.0%) or only with children (13.2%). Roughly the same proportion of households included two adults (22.7% two adults alone; 15.4% two adults and children). The remainder of households accommodated three or more adults (12.1% ≥ three adults alone; 7.3% ≥ three adults and children). The median levels of expenditure represented 13.8% (IQR 8.3–21.1%) of total reported household income across all types of household. The available data do not allow the accurate calculation of equivalised household income (by person), but similar proportions of household income were spent on tobacco in single *person* households (i.e., 1 adult no children: Median 17.2%; IQR 10.6–26.4%) and single *smoker* households (i.e., 1 adult with children: Median 15.5%; IQR 9.0–23.7%). The association between type of cigarette smoked (only FMC; only RYO; or both) and proportion of household income spent on tobacco was significant for each of the three household types described above (all households; single person; single smoker) (Table 5).

**Table 5:** Proportion of household income spent on tobacco by household type and tobacco type

	Median (IQR)				Kruskal-Wallis H by type of cigarette smoked	
Household	All smokers	Only smoke factory-made	Only smoke RYO	Smoke both	χ^2^(2)	*p*-value
All	13.8(8.3, 21.1)	17.3(10.4, 25.9)	9.8(6.5, 15.8)	12.1(6.9, 20.7)	67.27	*p* < 0.001
Single smoker	15.5(9.0, 23.7)	17.3(11.9, 26.4)	11.2(6.7, 15.8)	12.1(6.6, 21.1)	52.58	*p* < 0.001
Single adult	17.2(10.6, 26.4)	20.7(13.7, 26.4)	12.7(7.9, 17.2)	13.7(8.6, 26.4)	44.01	*p* < 0.001

Nearly half of the sample reported smoking induced deprivation (SID) in the past month (43.2%). Most (75.7%) either ‘agreed’ or ‘strongly agreed’ that spending money on cigarettes meant they were missing out on other important things. Binary SID was significantly associated with FSI (z = 17.076, *p* < 0.001), but not with cigarette type (χ^2^(2) = 3.134, *p* = 0.209).

## Discussion

When compared to the Australian population, a majority of FISCALS participants were at or below the 10th percentile of incomes [24]. They were also heavier smokers than the Australian smokers in the ITC Four Country Survey (HSI 3.0 c.f. 3.9 respectively) [25]. FISCALS participants endorsed each financial stress item at twice the rate over the past *month* as those from the lowest income quintile in HILDA in the past *12 months* [24]. They endorsed the first item of the scale (a single item measure of financial stress) three times more often than low income smokers from Australia, the UK, USA and Canada in the ITC sample (40.5% c.f., 13.6%) [21]. The rate of smoking induced deprivation reported by the FISCALS sample for the *past month* was four times that reported in the ITC survey for a *six-month* period. The data also reveal that over all household types tobacco expenditure accounted for 13.8% of total reported household income, and financial stress and deprivation were significantly associated.

Together these results suggest that heavy tobacco use, financial stress and deprivation are commonplace and interrelated. The data also clearly demonstrate that in our sample of low-income smokers tobacco was used despite an inability to meet financial obligations (e.g., paying bills) and smoking was given priority over other discretionary activities (e.g., leisure activities or holidays). However, high levels of motivation to quit indicated that our participants were seeking to change this situation.

Interestingly, neither (adjusted) financial stress nor deprivation were associated with increased motivation to quit or confidence in future quit attempt success. Thus, the experienced financial pressures and deprivation did not appear to increase cessation motivation or confidence in cessation success in the cohort. However, this may be due to the relatively high levels of financial stress and deprivation in this sample. That is, the lack of association may reflect difficulty in detecting effects due to a relative lack of variation in the sample, rather than to the absence of associations. It is also worth noting that the underlying causes of cessation motivation and confidence are complex, with a range of mechanisms related to both psychological and social factors. A 2010 meta-analysis of theory of planned behaviour [26] and smoking found that quit intention was most strongly related to perceived behavioural control, which may be associated with financial stress and deprivation. Further work is required to tease apart the complex patterns of associations and, potentially, causal relationships between the various psychological, social and environmental factors at play.

Increasing tobacco taxation is generally considered to be very successful way of reducing smoking in the population in general and in disadvantaged smoking populations in particular [3]. However, our data reveal that financial stress, a potential barrier to successful cessation [10, 27, 28], is highly prevalent in the FISCALS cohort of low income daily smokers. This creates a dilemma for policy makers: raising taxes may increase quit attempts, but the success of these attempts may be undermined if financial stress increases as the price rises. It will be important to put redistributive strategies in place to minimise the impact of cigarette price rises on poor smokers, perhaps by increasing subsidies for smoking cessation.

### Limitations

The main limitation of the study is that it relies upon self-report data collected using a CATI. Self-report data may be affected by exaggeration and or socially desirable responding. However this risk was minimal because the accuracy of responses was potentially verifiable and there was no incentive to respond in a socially desirable way [29].

A second limitation of this study may be that the inclusion and exclusion criteria for the FISCALS trial means that the smokers are unlikely to be representative of all low-income smokers in Australia. Their financial situation may be worse and their motivation to quit smoking higher. Even so we know that the cessation success rate for low SES smokers is generally low [3]. Thus, many will continue to live in the challenging financial situation described here.

## Conclusion

The data from FISCALS study provides a unique insight into the lives of a group of low income smokers who – like almost half of all low-income smokers in Australia – smoke more than 10 cigarettes per day. They were a group with high financial difficulty and declared motivation to quit, who had previously tried unsuccessfully to give up smoking. These results are consistent with those observed in other high cigarette tax environments [30] and suggest that low-income smokers will face increasingly difficult financial circumstances as tobacco products becomes less affordable. Furthermore, the effectiveness of tobacco price interventions may be undermined by the high prevalence of financial stress and smoking induced deprivation.

Our data reinforce the importance of researching and developing smoking cessation interventions that are responsive to the role that financial stress plays in reducing quit attempts and increasing relapse [11]. Actively managing the vicious cycle of financial stress and smoking may be required to reduce the increasing socioeconomic gradient in smoking prevalence in Australia and other high income countries.

## Additional file

Additional file 1: Fiscals Trial Enrolment. (TIF 1157 kb)

## References

[1] Australian Institute of Health and Welfare (2013). National Drug Strategy Household Survey in Drug statistics series.

[2] Hill S, Amos A, Clifford D, Platt S (2014). Impact of tobacco control interventions on socioeconomic inequalities in smoking: review of the evidence. Tob Control.

[3] Hiscock R, Bauld L, Amos A, Fidler JA, Munafo M (2012). Socioeconomic status and smoking: a review. Ann N Y Acad Sci.

[4] Bosdriesz JR, Willemsen MC, Stronks K, Kunst AE: Socioeconomic inequalities in smoking cessation in 11 European countries from 1987 to 2012. J Epidemiol Community Health 2015:jech-2014-205171. 10.1136/jech-2014-205171 25841241

[5] National Tobacco Strategy 2012–2018. Commonwealth of Australia: Intergovernmental Committee on Drugs Standing Committee; 2012.

[6] Syamlal G, Mazurek JM, Hendricks SA, Jamal A (2015). Cigarette smoking trends among US working adult by industry and occupation: findings from the 2004–2012 National Health Interview Survey. Nicotine Tobacco Res.

[7] Gartner CE, Barendregt JJ, Hall WD (2009). Predicting the future prevalence of cigarette smoking in Australia: how low can we go and by when?. Tob Control.

[8] Jha P (2015). Deaths and taxes: stronger global tobacco control by 2025. Lancet.

[9] Effectiveness of tax and price policies for tobacco control.. In IARC Handbooks of Cancer Prevention: Tobacco Control, vol. 14. Lyon: World Health Organization; 2011. 10.1136/tc.2010.039982 21115556

[10] Siahpush M, Spittal M, Singh GK (2007). Smoking cessation and financial stress. J Public Health.

[11] Siahpush M, Yong HH, Borland R, Reid JL, Hammond D (2009). Smokers with financial stress are more likely to want to quit but less likely to try or succeed: findings from the international tobacco control (ITC) four country survey. Addiction.

[12] Widome R, Joseph AM, Hammett P, Van Ryn M, Nelson DB, Nyman JA, Fu SS (2015). Associations between smoking behaviors and financial stress among low-income smokers. Preventive Medicine Reports.

[13] Siahpush M, Borland R, Scollo M (2003). Smoking and financial stress. Tob Control.

[14] Betzner A, Boyle RG, St Claire AW (2016). Price-minimizing behaviors in a cohort of smokers before and after a cigarette tax increase. Int J Environ Res Public Health.

[15] Martire KA, Mattick RP, Doran CM, Hall WD (2011). Cigarette tax and public health: what are the implications of financially stressed smokers for the effects of price increases on smoking prevalence?. Addiction.

[16] Courtney RJ, Bradford D, Martire KA, Bonevski B, Borland R, Doran C, Hall W, Farrell M, Siahpush M, Sanson-Fisher R (2014). A randomized clinical trial of a financial education intervention with nicotine replacement therapy (NRT) for low socio-economic status Australian smokers: a study protocol. Addiction.

[17] Borland R, Yong H-H, O'Connor R, Hyland A, Thompson M (2010). The reliability and predictive validity of the heaviness of smoking index and its two components: findings from the international tobacco control four country study. Nicotine Tobacco Res.

[18] Thomas D, Abramson MJ, Bonevski B, Taylor S, Poole SG, Weeks GR, Dooley MJ, George J (2015). Quitting experiences and preferences for a future quit attempt: a study among inpatient smokers. BMJ Open.

[19] Siahpush M, Carlin JB (2006). Financial stress, smoking cessation and relapse: results from a prospective study of an Australian national sample. Addiction.

[20] Pink B (2011). Household expenditure survey, Australia, 2009–10. Summary of results.

[21] Siahpush M, Borland R, Yong HH, Cummings KM, Fong GT (2012). Tobacco expenditure, smoking-induced deprivation and financial stress: results from the international tobacco control (ITC) four-country survey. Drug Alcohol Rev.

[22] Statistics ABo: Census of Population and Housing: Socio-Economic Indexes for Areas (SEIFA). (Statistics ABo ed. Australia; 2011.

[23] StataCorp: Stata Statistical Software: Release 14. vol. Release 14.1. College Station, TX: StataCorp LP; 2015.

[24] Summerfield M, Freidin S, Hahn M, Li N, Macalalad N, Mundy L, Watson N, Wilkins R, Wooden M (2014). HILDA user manual–release 13.

[25] Siahpush M, McNeill A, Borland R, Fong G: Socioeconomic variations in nicotine dependence, self-efficacy, and intention to quit across four countries: findings from the international tobacco control (ITC) four country survey. Tob Control 2006, 15:iii71-iii75. 10.1136/tc.2004.008763 PMC2593052 16754950

[26] Topa G, Moriano JA (2010). Theory of planned behavior and smoking: meta-analysis and SEM model. Substance Abuse and Rehabilitation.

[27] Reitzel LR, Langdon KJ, Nguyen NT, Zvolensky MJ (2015). Financial strain and smoking cessation among men and women within a self-guided quit attempt. Addict Behav.

[28] Caleyachetty A, Lewis S, McNeill A, Leonardi-Bee J (2012). Struggling to make ends meet: exploring pathways to understand why smokers in financial difficulties are less likely to quit successfully. Eur J Public Health.

[29] Chan D (2009). Statistical and methodological myths and urban legends: doctrine, verity and fable in the organizational and social sciences. So why ask me? Are self-report data really that bad.

[30] Farrelly MC, Nonnemaker JM, Watson KA (2012). The consequences of high cigarette excise taxes for low-income smokers. PLoS One.

[31] Lovibond PF, Lovibond SH (1995). The structure of negative emotional states: comparison of the depression anxiety stress scales (DASS) with the Beck depression and anxiety inventories. Behav Res Ther.

