# Supplementary figures and images for: Smoking and finances: baseline characteristics of low income daily smokers in the FISCALS cohort
Source: Int J Equity Health. 2017 Aug 30;16:157. doi: 10.1186/s12939-017-0643-6 (PMC5577825; doi:10.1186/s12939-017-0643-6)

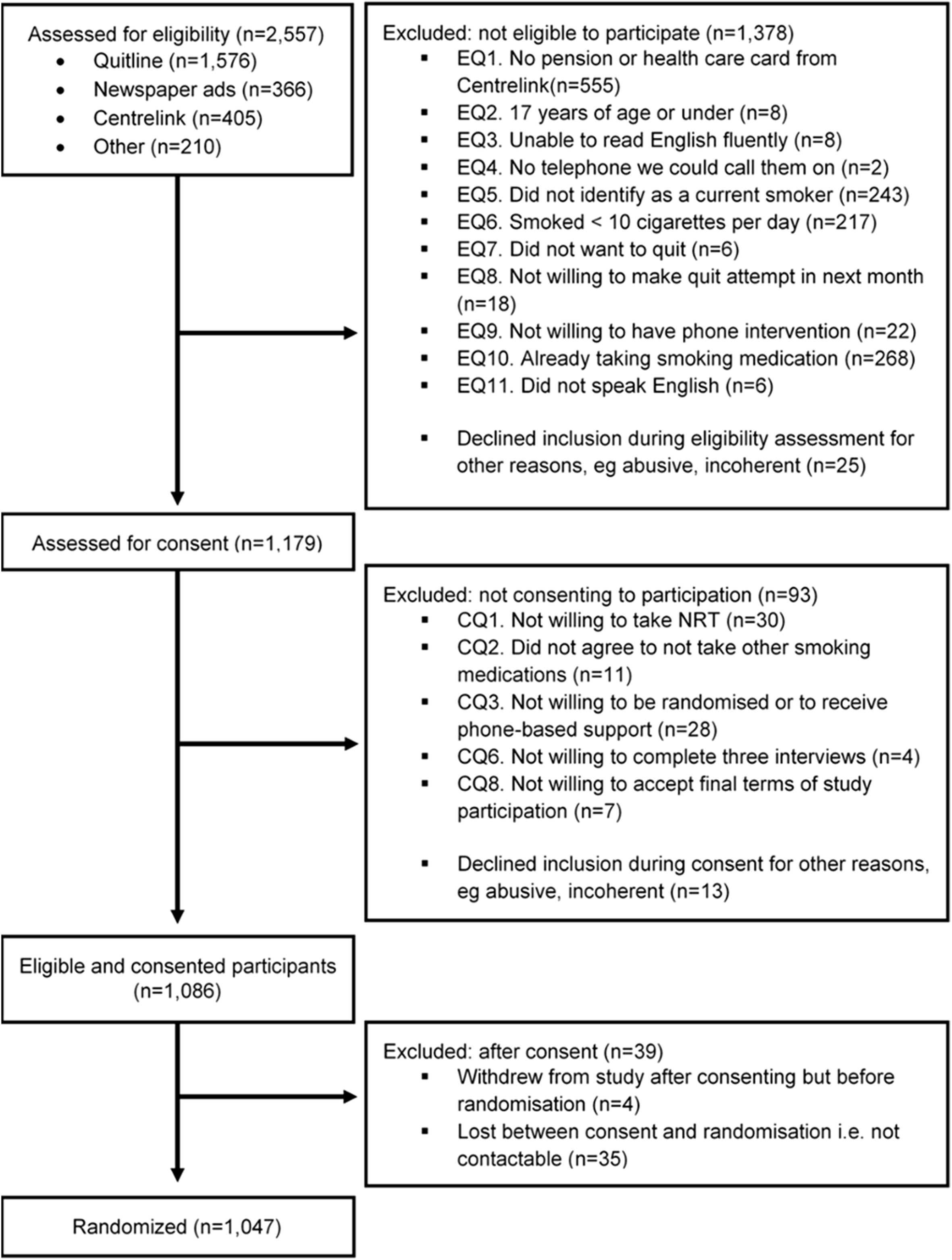

Supplement: Additional file 1: — Fiscals Trial Enrolment. (TIF 1157 kb) [file 12939_2017_643_MOESM1_ESM.tif]
